# Supplementary material for: Multiomics profiling of zebrafish embryonic cell line PAC2 across growth phases to assess its relevance for toxicological studies
Source: PLoS One. 2026 Apr 17;21(4):e0346109. doi: 10.1371/journal.pone.0346109 (PMC13089740; doi:10.1371/journal.pone.0346109)
Supplement: S12 Table — (DOCX) [file pone.0346109.s019.docx]

**S12 Table.** **Overview of expression in PAC2 cells of genes or proteins belonging to selected families related to toxicologically relevant processes and functions.** Selected gene families were checked by manual search in proteomics datasets (PDs) and transcriptomics dataset (TD) for respective keywords or gene symbols. *For SLC, only selected families known to be involved in transport of xenobiotics plus genes whose expression was detected on both the mRNA and protein level, are shown. Gene names shown in brackets ( ) indicate genes that were detected only in the TD’s counts list. Receptor names are indicated in square brackets [ ] if the identity is not obvious from gene name.

| **Gene group** | **Expressed on transcript level only** | **Expressed on protein level only** | **Expressed on both levels** |
| --- | --- | --- | --- |
| Fibroblast markers | (*vim*) |  | *cdh2*, *col1a1b*, *fn1a* |
| Epithelial markers |  |  | *cdh1*, *prr36b*, *sdc2*, *si:ch73-61d6.3*, *tjp2b* |
| Heat shock response and heat shock proteins | *hsf1*, *hsf2*, *(hsf5)*, *hsp70.1*, *hsp70.2*, *hsp70.3*, *(hsp70l)*, *hsp90aa1.2*, *hsp90b1*, *hspa12a*, *hspa13*, *hspa1b*, *hspa4a*, *hspa4b*, *hspa5*, *hspa8*, *hspa8b*, *hspa9*, *hspb1*, *hspb11*, *hspb2*, (*hspb6*), *hspb7*, *hspb8*, *hspb9*, *hspbap1*, *hspbp1*, *hspd1*, *hspe1*, *hsph1* | HSF, HSP1, HSP90A.1, HSP90A.2, HSPA4, HSPA4L | *hsf1*, *hsp90ab1*, *hspa12b*, *hspa14*, *hspg2* |
| Unfolded protein response | *xbp1*, *atf1*, *atf3*, *atf4a*, *atf4b*, *atf5a*, *atf5b*, *atf6*, *atf6b*, *atf7a*, *atf7b*, *atf7ip*, *atf7ip2* | - | *eif2a, eif2ak3 (perk), hyou1 (grp170), calr, calr3a, pdia7, txndc5* |
| Autophagy | *atg10*, *atg13*, *atg14*, *atg16l1*, *atg16l2*, *atg2a*, *atg4a*, *atg4c*, *atg4da*, *atg4db*, *atg5*, *atg9a*, *atg9b* | ATG7 | *atg3*, *atg101*, *atg12*, *atg4b, sqstm1 (p62)* |
| p53, response to hypoxia, metals and oxidative stress | *tp53*, *hif1ab*, *hif1al*, *mtf1*, *mtf2*, (*nfat5a*), (*nfat5b*), *nfatc1*, (*nfatc2a*), *nfatc2ip*, *nfatc3a*, *nfatc4*, *nfe2l1a*, *nfe2l1b*, *nfe2l2a*, *nfe2l2b*, *nfe2l3*, (*gpx3*), *hmox2a* | - | *blvrb, hif1an*, *nfatc3b*, *nqo1*, *sod1*, *sod2*, *gclc, gclm, gpx1a,* *gpx4b*, *gpx 7*, *gpx8*, *cat*, *hmox1a*, *hmox2b, gsr, prdx1, prdx2, prdx5, prdx6, txnrd3, fth1b* |
| ABC transporters | *abca1*, *abca2*, *abca3b*, (*abca5*), (*abcb11b*), *abcb6a*, *abcb6b*, (*abcc10*), (*abcc13*), (*abcc2*), *abcc4*, *abcc5*, (*abcc6a*), (*abcc9*), (*abcd1*), (*abcd2*), *abcd3b*, *abcd4*, *abcf2a*, (*abcf2b*), (*abcg1*) | ABCB6, ABCF2 | *abcb10*, *abcb6*, *abcb7*, *abcb8*, *abcc1*, *abcc3*, *abcc4*, *abcc5*, *abcc8*, *abcd3a*, *abce1*, *abcf1*, *abcf3*, *abcg2c* |
| CYP450s | (*cyp17a1*), *cyp17a2*, (*cyp19a1b*), *cyp1b1*, *cyp20a1*, (*cyp21a2*), *cyp26b1*, (*cyp26c1*), (*cyp27a1.1*), *cyp2aa1*, *cyp2aa12*, *cyp2aa8*, *cyp2ad3*, *cyp2j20*, *cyp2p10*, *cyp2v1*, (*cyp2x6*), (*cyp3a65*), *cyp46a1.3*, *cyp46a1.4*, *cyp4t8*, *cyp8b1.2* | CYP11C1, CYP2V2 | *cyp27a2*, *cyp2aa11*, *cyp2aa6*, *cyp2ae1*, *cyp46a1.2*, *cyp51*, *cyp8b1.3* |
| GSTs | (*gsta.2*), *gstcd*, *gstk2*, *gstk4*, *gstm.1*, *gstm.3*, *gsto2*, *gstp1.2*, *gstr*, *gstt1a*, *gstz1* | GSTM, GSTP1, GSTR1 | *gstk1*, *gstm.2*, *gstt2* |
| SULTs | (*sult1st2*), *sult2st1* |  | *sult1st1*, *sult2st3*, *sult3st1*, *sult6b1* |
| UGTs | *ugt2a1*, *ugt2a2*, *ugta2a3*, *ugt2a4*, (*ugt2a5*), *ugt2b1*, *ugt2b3*, *ugt5b3*, *ugt5b4*, *ugt5c3*, (*ugt8*) | UGT1B4 | *ugt1b1*, *ugt2b6*, *ugt5b1*, *ugt5b2*, *ugt5c1*, *ugt5c2*, *ugt5f1* |
| NATs | *nat8*, *nat8l2*, *nat9*, *nat10*, (*nat16*), *nat16l* | NAT1A | *nat14* |
| SLCs* | *slc10a3*, *slc10a7*, (*slc16a12a*), (*slc16a12b*), *slc16a13*, *slc16a1b*, *slc16a5a*, *slc16a6b*, (*slc16a9a*), *slc16a9b*, *slc22a15*, (*slc22a16*), *slc22a18*, *slc22a21*, (*slc22a23*), *slc47a1*, *slc47a3*, *slco1c1*, *slco2b1*, *slco3a1a*, (*slco3a1b*), *slco5a1a*, *slco5a1b* | SLC16A1, SLC16A2, SLC16A8, SLC22A31, SLC22A4 | *slc1a5*, *slc12a2*, *slc12a4*, *slc12a7a*, *slc12a7b*, *slc12a9*, *slc19a1*, *slc23a2*, *slc24a3*, *slc25a19*, *slc25a1a*, *slc25a1b*, *slc25a23a*, *slc25a24*, *slc25a26*, *slc25a35*, *slc25a37*, *slc25a40*, *slc25a46*, *slc25a5*, *slc25a51b*, *slc29a2*, *slc2a1b*, *slc30a5*, *slc30a6*, *slc30a7*, *slc30a9*, *slc35a2*, *slc35b1*, *slc36a1*, *slc36a4*, *slc38a10*, *slc38a5a*, *slc38a9*, *slc39a9*, *slc41a1*, *slc44a1*, *slc44a2*, *slc4a11*, *slc4a7*, *slc4a8*, *slc7a3b*, *slc7a5*, *slc9a6a* |
| MAPK, AKT and mTOR signaling | *mapk1*, *mapk3*, (*mapk4*), *mapk6*, *mapk7*, *mapk8a*, (*mapk8ip2*), *mapk13*, *akt2*, *akt3a*, *mtor*, *lamtor3*, *lamtor5* | MAPK14, AKT1, AKTIP | *mapk8b* (*jnk*), *mapk8ip3*, *mapk9* (*jnk*), *mapk11*, *mapk14a*, *mapk14b*, *mapkap1*, *akt3b*, *akt1s1*, *lamtor1*, *lamtor2*, *lamtor4* |
| Response to estrogens | *esr2a*, *esr2b*, *esrrb*, (*gper1*), *ebag9* | - | *esrra* |
| Response to corticoids | *nr3c2* [mineralocorticoid receptor], *gmeb1*, *gmeb2* | - | *nr3c1/fb13f09* [glucocorticoid receptor] |
| Response to aryl hydrocarbons | *ahr1b*, (*ahrrb*), (*arnt*), *aip*, (*nr1i2*) [PXR] | - | *ahr2*, *arnt2* |
| Interference with lipid metabolism | *pparab*, *pparda*, *ppardb*, *ppargc1a*, *ppargc1b*, *nr1h3* [LXRalpha], *nr1h4* [FXR] | FAM120B, FAM120C | - |
| Circadian rhythm genes | (*nr1d1*), *nr1d2a*, (*nr1d4b*), *aanat2*, *smad3*, *smad7*, *tgif1*, *tgfb2* | - | *smad3b*, *tgfb3* |
| Other nuclear receptors | (*nr0b1*), (*nr0b2a*), *nr1d2b*, (*nr1i2*), *nr2c1*, *nr2c2*, *nr2f1a*, (*nr2f1b*), *nr2f2*, (*nr2f5*), *nr4a1*, *nr4a2b*, *nr4a3*, (*nr5a1a*) | - | *nr2c2ap*, *nr2f6a*, *nr2f6b* |
